# “Simila Similibus Curantur,” or Like Is Cured by Like

**Published:** 1861

**Authors:** C. R. Parks


[The following article was received too late for insertion in the proper department.—EDS.]

### "SIMILIA SIMILIBUS CURANTUR," OR LIKE IS CURED BY LIKE.

READ BEFORE THE MCLEAN COUNTY MEDICAL SOCIETY.

BY C. R. PARKS, M. D.

*Mr. President:* In complying with the request of the McLean County Medical Society, I will state that my object is not to attack any particular theory of Medicine for the mere sake of making it appear absurd, but for the express purpose of ascertaining whether the above Homeopathic maxim has any foundation in truth. Tis truth we should aim at. I shall therefore endeavor to investigate this Homeopathic law, in an open, fair, candid, and I hope impartial manner, and see if it is in reality "one of the fixed laws of nature," as claimed.

The above *supposed law* is, together with "*infutesimal doses*," the sum and substance of the Homeopathist system of medicine—its real essence.

The question has been asked, How does it cure? In what way? "We believe—says a Homœopathic in reply—that the Homeopathic medical agent cures disease, *because the drug force in the agent is in relation of affinity—in the same way as the force in the magnet is to the iron—to the natural disease, superior to those which exists between the disease-producing agent and the physiologized tissues.*" If there is any meaning in this attempt at an explanation, I take it to be this: That there is a strong affinity or attraction existing between the "drug force" and the "natural disease," as in the case between the magnet and the iron, and that this affinity or attraction is stronger than that which exists between the disease-producing agent and the physiologieal tissues. This is the brittle thread upon which hangs the whole theory of the system of medicine called Homeopathy; that system which, if true, ought to do away with all the ills flesh is heir too, rejuvenate with most marvelous certainty, the *maid* of forty-five summers, and drive pains, like the fleeting vapor before a nor'-easter, just as well as to do what it now does—supply a means by which broken-down clerygymen and unsuccessful regulars and quacks may earn a livelihood with but little preparations, say a pamphlet on symptoms and a pocket-case of beautiful little pills such as are recommended for family use. Suppose there is a strong affinity between the "drug force" in the agent and the "natural disease," what does it amount to? And suppose it is stronger than that which exists between the "disease-producing agent" and the "physiological tissues," what better does that make it? Will the force in the agent with its affinity for the "natural disease" *draw* the *latter* to it as the *magnet does the iron?* Is such the idea? But 'tis acknowledged there is a lesser affinity between the disease-producing agent and the physiological tissues. What becomes of this affinity? It is causing disease, while the other is attracting that which it causes. Such being the case, 'tis easy to see there could be no cure. One agent is producing that for which another agent or force has a stronger affinity. Suppose a person is in the habit of using certain articles of diet, which invariably cause a superabundance of acid in the stomach. 'Tis known there is a strong affinity existing between acids and alkalines. We administer the latter and it neutralizes the former, but does it remove the *disease-producing agents* or *that which causes the acid?* Certainly not. Yet it does it, just as much, as the strong affinity which causes the agent to attract the disease, removes the affinity between the disease-producing agent and the physiological tissue, and no more. And yet such is the profound explanation of "Similia Similibus Curatunr."

What sense is there in the statement, that there is affinity between the "drug force" and "natural disease?" Disease "is a state of body in which the natural functions of the organs are interrupted or disturbed," "an opposite state to that of health." Would it not be much more in accordance with facts, to say that in curing disease the "drug force" operated upon the *disease-producing agent* or *that which brings about* or *continues* this "opposite state to that of health?"

We will now give our views of the vital forces controlling the economy, and state how these forces receive the material upon which they operate, and which in turn operates on them for good or for evil.

The laws of nature are fixed, so that in order to bring about any desired result, or avoid a calamity which follows the infraction of a fixed law, we must understand the true character and operation of the law. Some laws can be demonstrated. The operation and character of others are only inferented from legitimate deductions. If I can give a more rational explanation of the modus operandi of medicine in curing disease than the aforementioned *Homeopathic rationale*, and sustain it by incontrovertable examples, proving or going to prove its correctness, then will I have accomplished all I desire or expect —the vindication of *Regular Scientific Medicine.*

In the animal economy we have various systems, such as the nervous, muscular, arterial, glandular, &c., &c., each of which is presided over by a *force* or *law* of its own, or more properly speaking, forces of its own, as several forces are combined in the organic and functional arrangement of one of these systems. These different systems are specific in their purposes and aims. We will endeavor to elucidate our idea, by taking an example from the glandular system—the liver. There is in the liver a peculiar specific law for each different part and function in the organ, one for selecting material and manufacturing *lobules*, while others form within the lobule "a plexus of biliany ducts, a venus plexus, a continuation of the branches of the portal vein, hepatic vein, and of the minute arteries, veins and absorbents." There is a manufacturing law, as it were, for each of these different parts entering into and composing a lobules. The lobule having been constructed, there is an object, a destiny, a mission for it to fulfill. Another law then comes into active life and uses this lobule in a specific way to the accomplishment of this object or specific purpose. There is in fact a double purpose to be accomplished by this lobule, consequently there must be—many forces or modifications of this same force. "One separates the impurities from the venous blood of the chylo-poietic viscera, previously to its return into the general venous circulation, the other secretes a fluid necessary to chylification"—the bile.

As there is a force for construction, so is there one for destruction, breaking down in a peculiar way and casting off each lobule as soon as it has fulfilled its destiny. It will be understood then, that the same forces which enter into and control the simple lobule, are exactly similar to those controlling the various lobules composing the entire Hepatic Gland. They are all similar in their actions, but entirely independent of each other, comparatively speaking, for a portion of the gland may take on disease, while the remainder continues perfectly healthy.

There is always a cause for every condition even though the most skillful physician may not be able to detect it. My object now is to show, that although the whole number of lobules comprising the Hepatic Gland are each governed by precisely similar laws, yet when a certain cause is presented which tends to interfere with their normal healthy action, it is not equally attracted by the whole number, but only affects those into whose presence it is forced, and not by them attracted.

The forces act upon the material presented in the blood, out of which they construct their respective organs and parts of organs.

The healthy blood is composed of 110 to 140 parts of red corpuscules in 1000, 2.5 to 3.5 fibrine, 68 to 70 pure albumen, salt, etc., in small quantities—Andral.

When there is an excess or deficiency of these principles, it constitutes a morbid state of the blood. This blood is brought by means of the arterial and venous system within the influence of these forces, when they mutually commence moulding their respective organs, bone, muscle, nerve, etc., out of the few constituents entering into its composition, proving that the peculiarity is in the law, or how could the several different laws, make so many entirely different organs out of the same material.

The blood may be changed in various ways from this healthy standard. The lacteals are so organized and controlled as to be able to take up and throw into the circulation, various substances which are unnecessary for, and do not enter into the construction of an healthy organism, consequently there should be less difficulty,—in fact there is none—between this unnecessary material and the forces in the "physiological tissues," than between the latter and the necessary ingredients in the blood, or their force, so that the latter, by its superior affinity should according to the Homœopathic explanation entirely drive out and supersede the former.

This noxious element, be it miasmata or what not, is driven into the presence of these laws or vital force, when the effect is to depress some forces while others are excited to excessive action.

These laws are so constituted or vitalized as to be peculiarly influenced when certain drug forces, or any others, are forced upon them, or within the limits of their influence, or when one of the elements making up the tissue in which the force lives, is changed by the presence of a force acting chemically.

These forces being limited in their powers of endurance, may be depressed beyond the extent of that power, or excited to extinguishment. Then I say there is no "elective affinity" between the "disease producing agent" and the "physiological tissues," only when the agent acts chemically, 'tis forced upon them by surrounding circumstances over which they have no control. If then, the forces in the physiological tissues have no affinity for, but receive the disease producing agent under protest, (as seen by the pain, etc., it produces,) how can the Homœopathic explanation of the operation of their law, "similia, similibus, curantur" be true?

We conclude that they infer, that the drug force embodied in the remedial agent, is selected by the physiological tissue in preference to the disease producing agent—a kind of displacement, caused by the stronger affinity existing between the drug force and the force in the diseased tissue, than that which exists between the disease producing force, and the physiological tissues, consequently driving out the disease producing force. By this Homœopathic line of argument, they are led to assume, that two agents producing similar symptoms in an organ when brought within the influence of its laws—of attraction they say—separately, cannot produce their legitimate effects on the same tissues or forces at the same time conjointly. Is this true, that two forces acting similarly upon a physiological tissue when presented separately, when brought to bear conjointly, one will be selected to the exclusion of the other? I say no."

Suppose for example we combine three of the Hydragogue cathartics, ext. colocynth, ext. Jalaap and Gamboge. 'Tis known even to Homœopaths, (or ought to be,) that each of these drugs when given in certain quantities will produce copious watery stools. The drug force in the colocynth, acting on the force in the physiological tissues, produces this exhalation of watery fluid from the mucous membrane of the intestinal canal. A less dose will not produce this result. Exactly so is it with each of the other two drugs. Now suppose we combine the drugs, the quantity of each in the combination not being quite sufficient of itself to move the bowels when used alone. Does the force in the physiological tissue, select the one of the three drug forces, for which it has the strongest affinity, as the Homœopathic explanation of "similia similibus curantur" teaches? Certainly not. The drug forces are driven into the presence of the vital forces in the tissues, by the circulatory system, when each drug force produces its legitimate effect, and the result is a copious evacuation from the bowels. Is it not true then, and clear to the most obtuse comprehension, that two or more forces may, and in fact do, operate upon the physiological tissue at the same time aiding each other as it did here, in bringing about a certain result, instead of one being attracted by the physiological force or "natural disease" for which it had, as they say the stronger affinity, to the exclusion of the other.

Suppose a man is brought in with a frozen foot. What was in this case the disease producing agent? Cold of course.—Was there any *elective affinity*, between the force in the tissues of the foot, and the disease producing agent? We say none. In what way did the cold act to bring about this result? Simply through the law of the equalization of temperature by extracting the amount of heat in the foot, which was essentially necessary for the support of its vital forces, hence they cease to act, and the tissues are handed over to the law of decomposition.

But suppose a certain amount of heat has been left, so that the vital forces have not been entirely extinguished. In this case, according to the homœpathic law, we should select a remedy which when used in "massive doses on a healthy person" or part, will produce similar symptoms, and in this case they instance friction with the snow, cold water, etc. This I grant is good practice when properly used. But how does it cure the partially frozen tissues? Is it because it produces when used in "massive doses" on a healthy part, similar symptoms? Nonsense. Is it because of the stronger affinity which Homœopathists say exist between the force in the remedial agent and the diseased tissue or "natural disease?" How ridiculously absurd the idea. How does regular scientific medicine explain its action as a remedy? There is as has been stated a law of the necessary heat in the parts. The idea then is to impart the smallest quantity of heat to these enfeebled tissues and depressed vital forces in order to stimulate them as gently as possible, increasing the stimulus as the forces and tissues are able to bear it. Otherwise the heat would overcome the cohesive power of the forces over the tissues, and destroy them. Friction with snow, gets up, or brings out a certain amount of latent heat, but it will not do to continue this, we must next use cold water, and so graduate the amount of heat as to suit the condition of the parts. Soon nature will get up a reaction which may destroy the tissues by undue excitement, unless the excess is carried off by the proper means. 'Tis unnecessary to ask any reasonably intelligent, honest man, which is the more rational explanation of the modus operandi of the remedial agent.

Again. Suppose we apply a caustic substance to an organized tissue, what will be the immediate result? Chemical action. The caustic having a strong affinity for one or more of the elements entering into the composition of the tissue; overcomes the resistance of the vital force, which held the different elements together, and the result is the disorganization of the tissue. The disease producing agent in this case acts directly on the physiological tissue, and the destruction is complete or partial in proportion to the power of the caustic applied.

In the partial we find an enfeebled, depressed state of the forces in the tissues, caused by the limited chemical action of the caustic. This being the pathological condition of the parts, what are the therapeutical indications? "Similia similibus curantur" say they. Then they must use something which in "massive doses" will produce similar symptoms. Probably if I should advise a warm cataplasm to the part, they would say the heat in the cataplasm if applied in massive doses, would produce similar symptoms. But is that fact of itself any reason why it should cure the disease, or is it not merely a coincidence? The question to decide the matter is how does it cure or aid in bringing about, a healthy condition of the parts? Homœopathists say there is a stronger affinity existing between the "force in the remedial agent and the natural disease" than exists between the disease producing agent, and the physiological tissues. But in this case the disease producing agent has done its work and gone, leaving nothing but the effect, consequently we have their argument, or rather mere statement reduced to this—that there is a strong affinity existing between the heat in the cataplasm and the "natural disease," the inference being that this affinity brings about a cure. We desire to know in what particular way does it accomplish this cure? And their only reply is, "because of its stronger affinity," etc. The view we take of the modus operandi of the practice is that the moisture and heat stimulates greatly the depressed vital action in the partially decomposed tissues, until they assume their healthy state of action, while at the same time stimulates and assists the law of disintegration, thereby bringing about a more speedy separation of the dead from the living tissues.

One more example and we are done.

Suppose a Homœopathist is called in to see a patient laboring under the following symptoms: intense burning pain in the stomach, with violent irritation of the alimentary tube, purging and vomiting, corrosion of the mouth, tongue, etc.—Bloody evacuations from both stomach and bowels. He turns over to himself his supposed fixed law, "similia similibus curantur," and says, I must administer something which in massive doses on a healthy person will produce similar symptoms. God only knows what potency or dilution he might use if he confined himself closely to his own theory, in which case we feel assured of the fate of his victimized patient.—Having made up his mind what to give, some one suddenly informs the Homœopathic professor that the cause of the patient's sickness was a poisonous dose of corrosive sublimate (Hyo. Chlo. Corros.) Ah, "he exclaims," and what think you Mr. President becomes of his boasted theory and "fixed law." If he is not an ignoramus, it gives way to "massive doses," (not infinitesimal) of albumen—emesis having previously been assisted by the proper drugs or means—not because Albumen in the shape of white of eggs, will even produce similar symptoms under any circumstances, but because it has a strong affinity for the disease producing agent," converting the latter into a harmless compound. In this case where is their boasted affinity between the force in the albumen (remedial agent) and the "natural disease." Why would he administer sweet milk, flour, etc.? Whoever heard of their producing similar symptoms? On the contrary we desire to neutralize the cause and soothe the irritable mucus membrane of the bowels and stomach? Why do we give Hydrated proto-Sulphuret of Iron? Is it because its force has a strong affinity for the "natural disease," or is it not because of its strong affinity for the disease producing agent forming with it a harmless compound. Which is the more rational view of the modus operandi of the remedy in this case?

Would that all those who pretend to practice regular scientific medicine, were actuated by firmly benevolent motives, then would they investigate the science out of a desire to know and be able to vindicate its excellence. Alas too many are willing to go forth to the world with but a faint idea of scientific medicine. Just knowledge enough to enable them to benefit themselves pecuniarily. This is the same spirit which actuates the Quack in getting up his new nostrum—in fact it is quackery, and from this class all the isms in medicine spring—also the visionary man of one idea who would have us all believe that a wet sheet is a panacea for every ill. All of us who love regular scientific medicine on account of the blessings it confers upon suffering humanity, should cling to it with jealous care and affection, that even some of its professed friends as well as its most bitter enemies shall not stab it in the dark. Let the midnight watch tell of our devotion to its truths, and our desire to know more of them, and God forbid that that watch or any other, should ever find us concocting some new theory through which we might draw in the visionary and unsuspecting for the mere purpose of gain.

Bloomington, Ill., July 8th, 1861.

From Minutes.—On motion of Dr. S. Noble, a vote of thanks was tendered Dr. Parke for his contribution and a copy asked for publication in the Chicago Medical Journal.

*The Great Philanthropist Sylvester Lind.*—Most of our readers may have heard of the Lind University and the great medical reform department established by Mr. Lind with worthy co-operators. The following extracts from the Chicago *Daily Tribnne* of Thursday, June 27, 1861, exhibits a few only of the facts which the public ought to know, concerning the source of the funds with which Mr. Lind may have been able to reform and convert the world medical and non-medical: "It is only a matter of common fairness to state that the diversion and misappropriations (of the city funds) are laid solely to the act of its Treasurer, Sylvester Lind." "It is not a pleasant reflection that a man who could endow a sectarian institution of learning to the amount of $100,000, should by his official misconduct cause the indefinite suspension of our public schools."

The amount of funds in the hands of the Board of Sewerage of the city of Chicago, according to the report of the City Controller, S. D. Ward, Esq., published in the *Tribune* of the same date, is $158,215.12.

If any one wishes to know where the balance of the fund to establish the "Lind University," after deducting the loss on the above, might come from, let him enquire about the streets, as we are not disposed to go beyond the statement of the *Tribune*, which has heretofore belonged to the same political party with "Brother Johnny," and cannot be suspected of a disposition to exaggerate.

---

*Dr. Douglas' Case of Spina Bifida.*—A subsequent number of the *Pacific Journal* states that the case mentioned on pages 394 and 395 of this journal resulted in a perfect cure without further difficulty.

